# Whole Genome Characterization, Phylogenetic and Genome Signature Analysis of Human Pandemic H1N1 Virus in Thailand, 2009–2012

**DOI:** 10.1371/journal.pone.0051275

**Published:** 2012-12-12

**Authors:** Jarika Makkoch, Kamol Suwannakarn, Sunchai Payungporn, Slinporn Prachayangprecha, Thaweesak Cheiocharnsin, Piyada Linsuwanon, Apiradee Theamboonlers, Yong Poovorawan

**Affiliations:** 1 Center of Excellence in Clinical Virology, Faculty of Medicine, Chulalongkorn University, Bangkok, Thailand; 2 Department of Biochemistry, Faculty of Medicine, Chulalongkorn University, Bangkok, Thailand; The University of Hong Kong, China

## Abstract

**Background:**

Three waves of human pandemic influenza occurred in Thailand in 2009–2012. The genome signature features and evolution of pH1N1 need to be characterized to elucidate the aspects responsible for the multiple waves of pandemic.

**Methodology/Findings:**

Forty whole genome sequences and 584 partial sequences of pH1N1 circulating in Thailand, divided into 1^st^, 2^nd^ and 3^rd^ wave and post-pandemic were characterized and 77 genome signatures were analyzed. Phylogenetic trees of concatenated whole genome and HA gene sequences were constructed calculating substitution rate and d_N_/d_S_ of each gene. Phylogenetic analysis showed a distinct pattern of pH1N1 circulation in Thailand, with the first two isolates from May, 2009 belonging to clade 5 while clades 5, 6 and 7 co-circulated during the first wave of pH1N1 pandemic in Thailand. Clade 8 predominated during the second wave and different proportions of the pH1N1 viruses circulating during the third wave and post pandemic period belonged to clades 8, 11.1 and 11.2. The mutation analysis of pH1N1 revealed many adaptive mutations which have become the signature of each clade and may be responsible for the multiple pandemic waves in Thailand, especially with regard to clades 11.1 and 11.2 as evidenced with V731I, G154D of PB1 gene, PA I330V, HA A214T S160G and S202T. The substitution rate of pH1N1 in Thailand ranged from 2.53×10^−3^±0.02 (M2 genes) to 5.27×10^−3^±0.03 per site per year (NA gene).

**Conclusions:**

All results suggested that this virus is still adaptive, maybe to evade the host's immune response and tends to remain in the human host although the d_N_/d_S_ were under purifying selection in all 8 genes. Due to the gradual evolution of pH1N1 in Thailand, continuous monitoring is essential for evaluation and surveillance to be prepared for and able to control future influenza activities.

## Introduction

Since the United States Center of Disease Control and Prevention (US-CDC) have launched the report on a confirmed case of human pandemic influenza virus (pH1N1) infection in a child in southern California on April 21, 2009, [1] this virus has spread on a global scale. The Center for Disease Control and Prevention provided an updated (September 11, 2009) situation report on the international cases stating that there were over 227,607 laboratory-confirmed cases of human pandemic influenza virus H1N1 infection with at least 3,205 deaths worldwide [2]. In Thailand, the first two cases of human pandemic influenza H1N1 infection were reported by the Bureau of Emerging Infectious Diseases, Department of Disease Control, Thai Ministry of Public Health on May 12, 2009 [3].This virus can be transmitted from person to person via droplet transmission [4]. Therefore, the virus can be spread easily in overcrowded areas. The symptoms associated with H1N1 infection are usually less severe than those of seasonal influenza A virus infection. Most patients experience only mild to moderate symptoms, such as high body temperature in conjunction with some respiratory symptoms such as cough, sore throat and headache. Some people may have severe symptoms like pneumonia and acute respiratory distress syndrome [5]. In addition, individuals with underlying complications such as heart disease, chronic lung disease, or diabetes potentially display more severe symptoms [6].

Many previous studies have provided genetic analysis of pH1N1 indicating that this virus is composed of NA (neuraminidase) and M (matrix) genes originating from the Eurasian swine lineage while the other six gene segments have been traced back to a triple-reassorted North American swine lineage. Specifically, three polymerase genes, PB2 (basic polymerase 2), PB1 (basic polymerase 1) and PA (acid polymerase) are combinations of the North American swine, avian and human H3N2 lineages while the HA (hemagglutinatin), NP (nucleoprotein) and NS (non-structural) genes belong to the classical swine lineage [7]–[10]. Moreover, several reports have identified the genome signatures and point mutations significant for improved fitness and transmission capacity among humans [11]–[13]. For example, mutations in the NS1 and PB1-F2 genes affect viral pathogenicity, mutations on the sialic acid binding site of the antigenic HA molecule determine host range of pH1N1 and mutations occurring in the NA gene represent antiviral drug susceptibility. In response to this pandemic, attempts in both hemispheres have been intensified at predicting the emergence of circulating influenza viruses in order to develop vaccines. From September 2010 to January 2011, the major strains of H1N1 influenza virus infecting humans were pH1N1. Hence, WHO included the vaccine virus A/California/7/2009 in the recommended composition of influenza virus vaccines for the northern hemisphere in 2012–2013 [14]. However, monitoring adaptive mutations of pandemic influenza virus should be performed continuously to elucidate the evolutionary trend for vaccine design.

As in our previous study, the trend of confirmed cases of pH1N1 infection demonstrated that there were 3 dominant waves of outbreaks of pH1N1 virus in Thailand from the first outbreak in 2009 until 2012, with the first peak from June to October 2009, the second wave from December 2009 to March 2010, and the third wave from July to November 2010. In this study, we performed whole genome characterization to describe the evolutionary trend of human pandemic influenza virus which has been circulating in Thailand during three waves and evaluated the potential positions of mutated residues and their contribution to virulence and pathogenesis of pH1N1 to provide vital information required for preparing control strategies, such as defining vaccine composition and efficiently predicting the potentially emerging strains in a future wave of outbreak.

## Results

### Study population

The study population consisted of 11,753 specimens comprising 5,783 males and 5,970 females (male∶female ratio = 1∶1.03), with their age ranging from 1 month to 86 years (mean age = 35.7 years). All collected samples were divided into samples isolated during the first wave (W1) from June to October 2009 (2,417 specimens), the second wave (W2) from December 2009 to March 2010 (1,250 specimens), the third wave from July to November 2010 (2,982 specimens) and post-pandemic period (PP) which started after November 2010 (3,113 specimens). The pH1N1 virus circulating between W1 and W2 was categorized as interwave 1 (IW1; 181 specimens) and those circulating between W2 and W3 were categorized as interwave 2 (IW2; 1,810 specimens). Upon sub-typing by real-time RT-PCR, 43.94% (5,164 specimens) provided negative results, 28.32% (3,329 specimens) were identified as pH1N1 influenza virus infection, 20.55% (2,415 specimens) as H3 seasonal influenza virus infection, 0.05% (692 specimens) as influenza B virus infection, 4 specimens as seasonal H1N1 virus infection and 0.01% (149 specimens) were inadequately collected specimens.

Forty samples positive for pH1N1 virus were randomly selected for whole genome characterization in chronological order from May 2009 to April 2012 and 584 partial sequences were randomly selected for analysis of the 7 major mutations of 6 genes (92, 92, 92, 93, 93, and 122 sequences for PB2, PB1, HA, NA, M and NS respectively). Each isolate represented a different geographical location and disease severity. All the whole genome sequences and partially selected for 7 major mutations sequences were submitted to GenBank database. All accession numbers of whole genome sequences and partial genome sequences were listed in Table S2 and S3 respectively. The details of each patient selected for whole genome characterization were listed in Table 1.

**Table 1:** Details of patients and pH1N1 influenza virus isolated in Thailand from May 2009 to April 2012.

No.	Name of Isolate	Date of Collection	Location	Age (yr)	Sex	Disease Severity**
**1**	A/Thailand/nonthaburi102/2009	5/2009	Bangkok		M	Acute
**2**	A/Thailand/104/2009	5/2009	Bangkok		M	Acute
**3**	A/Thailand/CU-B5/2009	13/6/2009	Bangkok	12	M	Mild
**4**	A/Thailand/CU-H9/2009	17/6/2009	Bangkok	14	M	Mild
**5**	A/Thailand/CU-H106/2009	2/7/2009	Bangkok	12	F	Mild
**6**	A/Thailand/CU-H276/2009	22/7/2009	Bangkok	17	F	Severe
**7**	A/Thailand/CU-H340/2009	5/8/2009	Nakorn Sri Thammarat	70	M	Severe, Acute
**8**	A/Thailand/CU-C161/2009	26/8/2009	Khon Kaen	23	M	Moderate
**9**	A/Thailand/CU-H567/2009	2/9/2009	Bangkok	24	M	Mild
**10**	A/Thailand/CU-H572/2009	3/9/2009	Nakorn Sri Thammarat	7	F	Mild
**11**	A/Thailand/CU-H847/2009	22/10/2009	Bangkok	28	F	Mild
**12**	A/Thailand/CU-H910/2009	9/11/2009	Bangkok	13	M	Mild
**13**	A/Thailand/CU-C602/2010	26/1/2010	Khon Kaen	7	F	Mild
**14**	A/Thailand/CU-B938/2009	18/7/2009	Bangkok	18	M	Mild
**15**	A/Thailand/CU-H1222/2010	13/1/2010	Bangkok	28	M	Mild
**16**	A/Thailand/CU-H1255/2010	18/1/2010	Bangkok	2	F	Mild
**17**	A/Thailand/CU-H1818/2010	18/1/2010	Bangkok	35	F	Mild
**18**	A/Thailand/CU-H1821/2010	31/3/2010	Bangkok	30	F	Mild
**19**	A/Thailand/CU-H1786/2010	20/3/2010	Bangkok	47	M	Severe
**20**	A/Thailand/CU-B2357/2010	20/4/2010	Bangkok	4	M	Mild
**21**	A/Thailand/CU-B2417/2010	5/7/2010	Bangkok	53	F	Moderate
**22**	A/Thailand/CU-B2543/2010	6/8/2010	Bangkok	21	M	Mild
**23**	A/Thailand/CU-H2176/2010	17/8/2010	Bangkok	N/A	F	Mild
**24**	A/Thailand/CU-H2358/2010	8/9/2010	Bangkok	6	F	Mild
**25**	A/Thailand/CU-H2283/2010	31/8/2010	Bangkok	14	F	Moderate
**26**	A/Thailand/CU-H2389/2010	10/9/2010	Bangkok	6	M	Mild
**27**	A/Thailand/CU-H2911/2011	20/1/2011	Bangkok	33	F	Mild
**28**	A/Thailand/CU-C1157/2010	7/9/2010	Khon Kaen	14	F	Mild
**29**	A/Thailand/CU-H2548/2010	27/9/2010	Bangkok	5	M	Mild
**30**	A/Thailand/CU-B4339/2010	2/11/2010	Bangkok	1	F	Mild
**31**	A/Thailand/CU-B4148/2010	9/10/2010	Bangkok	5	M	Moderate
**32**	A/Thailand/CU-H2698/2010	3/11/2010	Bangkok	4	F	Mild
**32**	A/Thailand/CU-B4656/2011	1/3/2011	Bangkok	N/A	M	Mild
**34**	A/Thailand/CU-B4662/2011	15/6/2011	Bangkok	1	M	Mild
**35**	A/Thailand/CU-B4717/2011	20/5/2011	Bangkok	19	F	Mild
**36**	A/Thailand/CU-B4773/2011	15/6/2011	Bangkok	25	M	Mild
**37**	A/Thailand/CU-B5356/2011	18/8/2011	Bangkok	36	M	Mild
**38**	A/Thailand/CU-B5515/2011	27/8/2011	Bangkok	28	M	Mild
**39**	A/Thailand/CU-B6181/2012	7/4/2012	Bangkok	38	M	Mild
**40**	A/Thailand/CU-B-6213/2012	29/4/2012	Bangkok	22	F	Mild

** Mild = Out patient, Moderate-In patient or admitted cases. Severe = Patient who treated in intensive care unit.

### Phylogenetic Analysis

**Figure 1:**
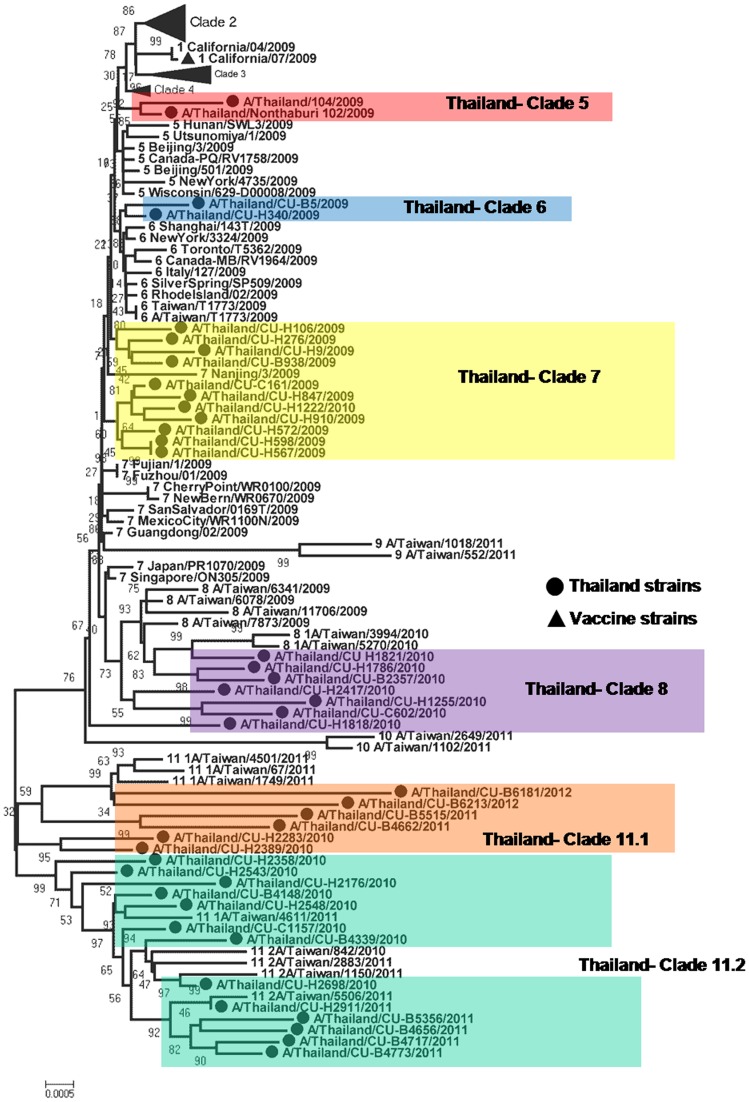
Phylogenetic tree of concatenated sequences of pH1N1 influenza viruses circulating in Thailand during 2009–2012.

**Figure 2:**
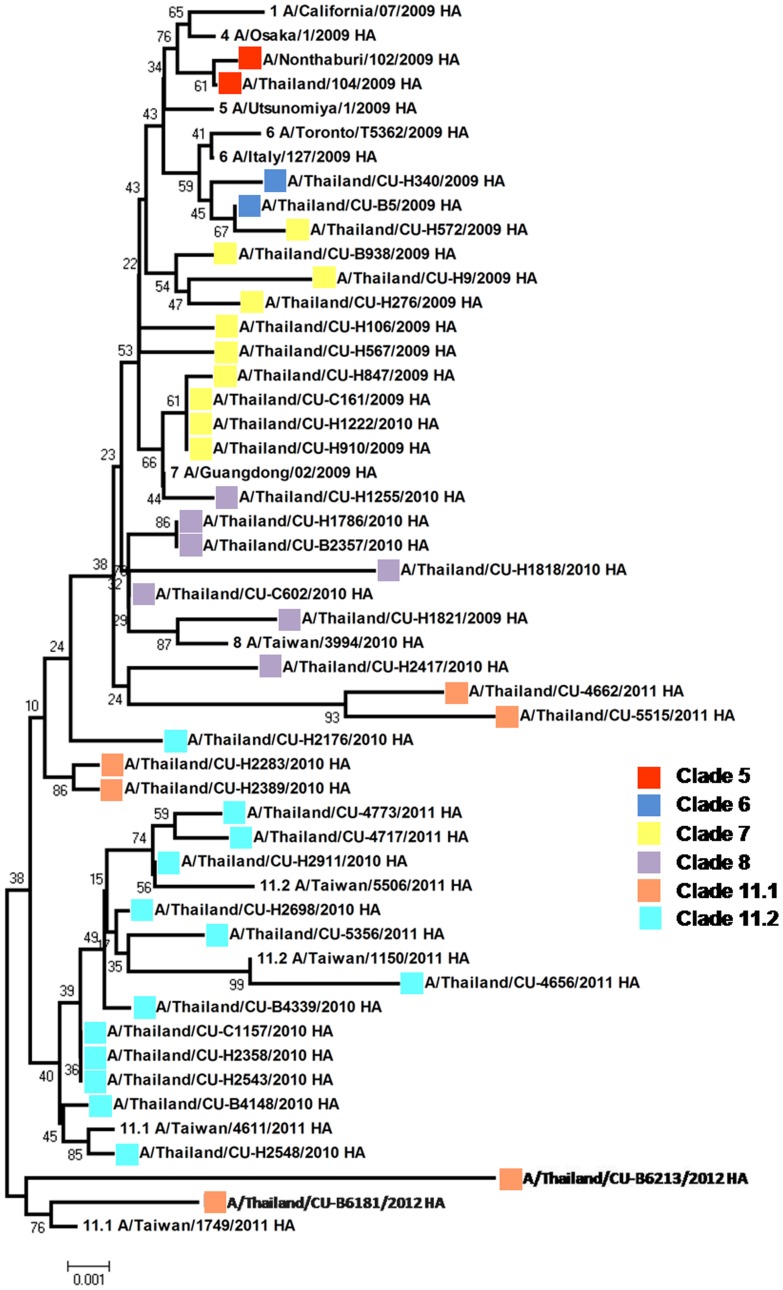
Phylogenetic tree of the HA gene of pH1N1 influenza viruses circulating in Thailand during 2009–2012.

Whole genome sequence analysis of 40 pH1N1 isolates in Thailand revealed different maximum Kimura distances between nucleotides ranging from 1.03% (M) to 1.85% (NS1) with percentages of amino acid divergence in each gene segment ranging from 1.26% (M) to 3.41 (HA) (Table 2). Phylogenetic analysis of the 40 concatenated whole genome sequences was performed along with 250 concatenated genomes of global isolates from previous studies [15], [16], [17]. The bootstrap supported phylogenetic tree showed that pH1N1 isolates from three waves in Thailand clustered into 6 distinct clades, namely, clades 5, 6, 7, 8, 11.1 and 11.2 (Fig. 1). The phylogenetic tree of the full-length HA gene was constructed using all isolates from Thailand and 11 representative isolates selected from each HA clade viruses as references (Fig. 2). The results showed that the first two isolates from May, 2009 belonged to clade 5 and pH1N1 viruses isolated during W1 and IW1 (from June to October 2009) were categorized into clades 5, 6 and 7 while pH1N1 viruses isolated during W2 and IW2 were classified into clades 7 and 8. The HA tree topologies showed a pattern similar to that in the concatenated tree. The phylogenetic results showed that in the first pandemic wave during the rainy season in Thailand, clade 7 was predominant (69.23%), followed by clade 8 which prevailed during the second pandemic wave in Thailand (83.33%) from winter until beginning of summer. Finally, the pH1N1 viruses circulating in W3 and PP during the rainy season of 2011 to 2012 belonged to clades 8, 11.1 and 11.2 with different proportions (5, 30 and 65% respectively).

**Table 2:** Diversity percentages, selection pressure and substitution rates of 40 whole genome sequences of pH1N1 virus circulating in Thailand from May 2009 to April 2012.

Gene	Max. Kimura distance as % (nt)	Max. distance as % (aa)	Omega (*d*N/*d*S)	Mean substitution rate (subst. per site year −1)×10−3 (±SD)
**PB2**	1.15	2.92	0.206	3.47±0.01
**PB1**	1.38	2.70	0.156	3.34±0.02
**PA**	1.37	2.06	0.242	4.03±0.01
**HA**	1.67	3.41	0.324	5.18±0.01
**NP**	1.22	1.42	0.089	3.01±0.02
**NA**	1.22	2.16	0.449	5.27±0.03
**M**	1.03	1.26	-	3.02±0.02
**M1**	1.20	1.61	0.181	3.03±0.02
**M2**	1.38	3.14	0.634	2.53±0.06
**NS**	1.58	2.59	-	4.41±0.03
**NS1**	1.85	2.78	0.337	4.73±0.03
**NS2**	1.58	2.59	0.534	4.40±0.04

### Substitution rates and selection pressure

The 40 whole genome sequences of pH1N1 virus circulating in Thailand during 2009–2012 were compared with the common reference sequence A/California/07/2009 from the GenBank database to calculate nucleotide substitution rates for each gene (Table 2). The mean substitution rates per gene segment ranged from 2.53×10^−3^±0.02 (for the M2 genes) to 5.27×10^−3^±0.03 (for the NA gene) substitutions per site per year. For the adaptive molecular evolution assessment of pH1N1, overall selective pressure operated on the determinant encoding each gene segment of pH1N1 virus were analyzed by estimating the ratio of non-synonymous (*d*_N_) to synonymous (*d*_S_) substitutions (ω = *d*_N_/*d*_S_) across the lineages on a codon by codon basis. Selective pressure was defined as follows: ω = 1 indicates neutral evolution, ω<1 indicates negative or purifying selective pressure, and ω>1 indicates positive selection. In Thailand, the pH1N1 genes were under purifying selective pressure ranging from ω values (*d*_N_/*d*_S_) = 0.089 (for the NP gene) to 0.634 (for the M2 gene) (Table 2).

### Genome signature analysis of pH1N1 virus isolated during 2009–2012 in Thailand

**Figure 3:**
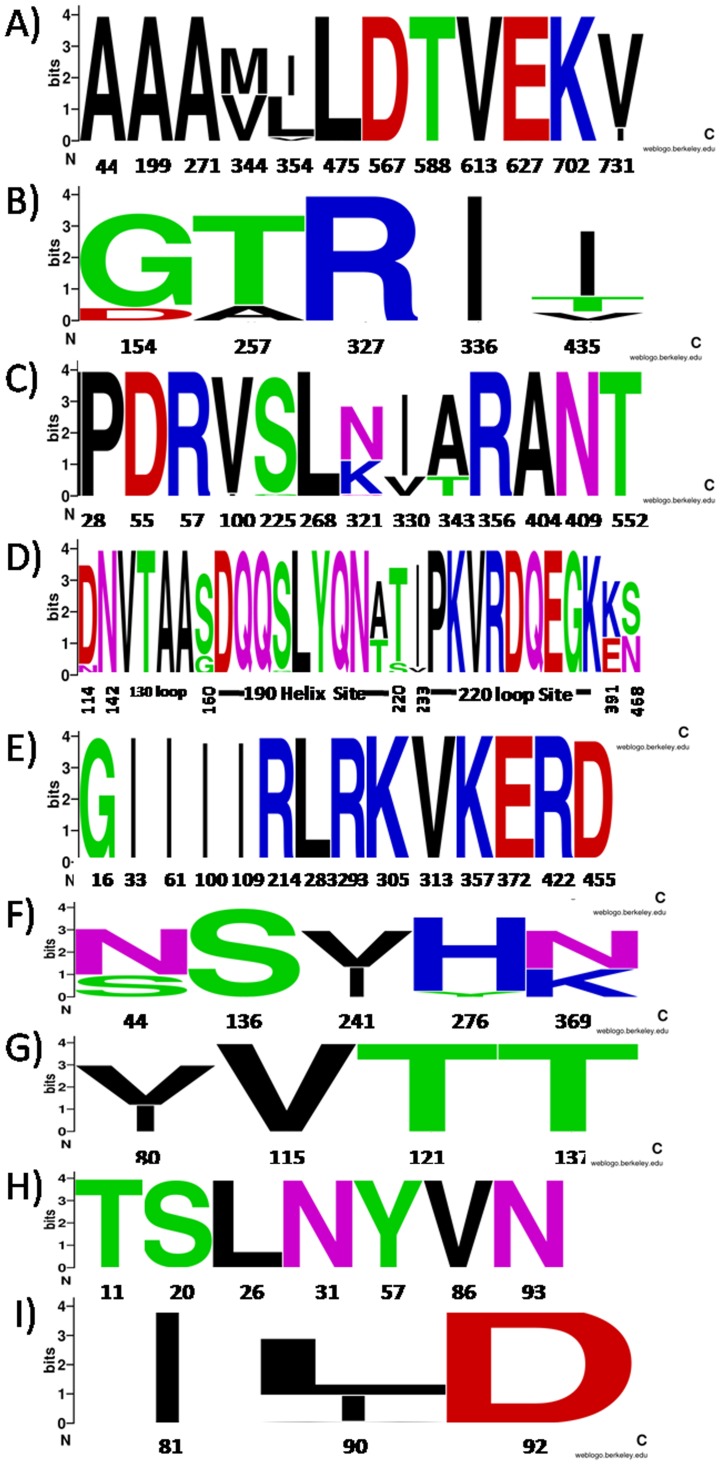
Genome signatures in amino acid residues of human pandemic H1N1 in Thailand during 2009–2011 in gene; A) PB2, B) PB1, C) PA, D) HA, E) NP, F) NA, G) M1, H) M2 and I) NS. The graphical presentation was constructed using WebLogo. The height of symbol indicates the relative frequency of the corresponding amino acid at that position. Residue positions are given based on the nucleotide positions of each gene.

The 40 full-length sequences and 584 partial sequences of pH1N1 influenza virus circulating in Thailand from May 2009 to April 2012 were analyzed for 77 point mutations described in previous studies [11]–[13], [15]–[19]. Many amino acid substitutions were identified during the evolution of pH1N1 in Thailand (Fig. 3 A–I, Table 3 and 4).

**Table 3:** Amino acid residues of whole genome sequences of human pandemic H1N1 influenza virus strains at 77 positions separated by genes.

Gene	Position	pH1N1 residue	Major %
**PB2**	44	**A** (40)	100
	199	**A** (40)	100
	271	**A** (40)	100
	344	**V** (22), **M** (18)	55.00
	354	**I** (14), **L** (24), **V** (2)	36.84
	475	**L** (40)	100
	567	**D** (40)	100
	588	**T** (40)	100
	613	**V** (40)	100
	627	**E** (40)	99.50
	702	**K** (40)	100
	731	**V** (35), **I** (5)	87.50
**PB1**	12	**Stop** (40)	100
	154	**G** (35), **D** (5)	87.50
	257	**T** (40)	100
	327	**R** (40)	100
	336	**I** (40)	100
	435	**I** (28), **V**(6), **T** (6)	73.68
**PA**	28	**P** (40)	100
	55	**D** (40)	100
	57	**R** (40)	100
	100	**V** (39), I (1)	97.50
	225	**S** (39), **G** (1)	97.50
	268	**L** (40)	100
	321	**N** (25), **K** (14), Q (1)	62.5
	330	**I** (32), **V** (8)	80.00
	343	**A** (32), **T** (8)	80.00
	356	**R** (40)	100
	404	**A** (40)	100
	409	**N** (40)	100
	552	**T** (40)	100
**HA**	114	**D** (36), **N** (4)	90.00
	142	**N** (40)	100
	135–138	**VTAA** (40)	100
	160	**S** (32), **G** (8)	80.00
	190–198	**DQQSLYQNA (40)**	100
	214	**A** (25), **T** (15)	62.5
	220	**S** (5), **T** (35)	87.50
	221–228	**PKVRDQEG** (40)	100
	233	**I** (37), **V** (2), T (1)	92.50
	300	**K** (39), **E** (1)	97.50
	391	**E** (16), **K** (24)	60.00
	468	**S** (23), **N** (17)	57.50
**NP**	16	**G** (40)	100
	33	**I** (40)	100
	61	**I** (40)	100
	100	**V** (1), I (39)	97.50
	109214	**I** (39), L (1)**R** (40)	97.50100
	283	**L** (40)	100
	293	**R** (40)	100
	305	**K** (40)	100
	313	**V** (40)	100
	357	**K** (40)	100
	372	**E** (40)	100
	422	**R** (40)	100
	455	D (39), E (1)	97.50
**NA**	44	**N** (27), **S** (13)	67.50
	136	S (40)	100
	241	**V** (22), **I** (18)	55.00
	276	**H** (37), **Y** (3)	92.50
	369	**N** (23), **K** (17)	57.50
**M1**	80	**V** (24), **I** (16)	60.00
	115	**V** (40)	100
	121	**T** (40)	100
	137	**T** (40)	100
**M2**	11	**T** (40)	100
	20	**S** (40)	100
	26	**L** (40)	100
	31	**N** (40)	100
	57	**Y** (40)	100
	86	**V** (40)	100
	93	**N** (40)	100
**NS1**	81	**I** (40)	100
	90	**L** (27), **I** (12), **V** (1)	67.50
	92	**D** (40)	100
**NS2**	107	**L** (40)	100

**Table 4:** Amino acid residues of partial sequences of human pandemic H1N1 influenza virus strains at 7 positions separated by genes.

Gene	Position	pH1N1 residue	Major %
**PB2**	627	D(91/92), E(1/92)	98.91
**PB1**	12	Stop (92/92)	100
**HA**	221–228	PKVRDQEG (92/92)	100
**NA**	47–68	No deletion (93/93)	100
	136	S (93/93)	100
**M2**	31	N (93/93)	100
**NS1**	92	D (122/122)	100

## Discussion

Due to the lack of genetic reassortment and similar tree topologies of each gene segments, concatenated phylogenetic tree was justified to be used for evolutionary analysis of pH1N1 influenza virus to exaggerate phylogenetic resolution of this virus, as previous studies [15], [16]. Phylogenetic analysis of the concatenated whole genome of pH1N1 showed absence of any distinct geographical distribution, but a pattern of chronological distribution. During 2009–2012, there were 3 waves of human pandemic influenza virus in Thailand, which has a climate of tropical savannah, with summer from mid February to mid May, rainy season from mid May to October and winter from November until mid February [20]. Based on the similar evolutionary trend of the whole genome tree and HA tree, pH1N1 isolates circulating in the rainy season of 2009 belonged to clades 5, 6 (which emerged from a common node) and clade 7 while those circulating from winter to summer 2010 were for the most part in clade 8. However, the results indicated that the pH1N1 virus has distinctively adapted and shared many substitutions. This phenomenon may indicate the founder effect during the summer and rainy season of 2010. Many factors could be responsible for this profound genetic distinction, such as the beginning of the school semester, the high season of tourism in Thailand, or quantity of antiviral drug use in Thai population. Although there were different sublineages of pH1N1 virus, the low maximum distance indicated that new variants were still closely related to the prior sublineages and the pH1N1 vaccine could be effective in this region.

### Substitution rate and selection pressure

In Thailand, all pH1N1 genes were under purifying selection with 0.089 (NP gene)≤ω≤0.634(M2 gene), indicating a likelihood of 9–63% non-synonymous mutation compared to synonymous mutation [21]. However, these evolutionary ratios were relatively high compared to those in human seasonal H1N1 and H3N2 [22], archived as evolutionary stasis, based on the very low rates of amino acid change (ω) in each gene. [23]. The higher selective pressure and substitution rates in some genes, such as HA, NA M2 and NS could be described by the change of amino acids in these genes of pH1N1 virus has been adapted to a new host species or avoid a host's immune response [24]. Therefore, it seems plausible to suggest that this pH1N1 virus will tend to stay and adapt for a longer period in the human host before it will become seasonal influenza and thus, more static. The evolutionary rates should be continuously monitored and compared to the epidemiological study. The other possible aftermaths of year-to-year divergence in host species which should be considered are niche differentiation and character displacement. This divergence described an accumulation of antigenic drift of influenza virus overtime where virus changes it pathogenic epitopes to escape host immune system due to host immunological pressure. This phenomenon resulted in viral persistence in host population or the alteration to expand viral niche which can create the resource partitioning, such as changing tissue tropism and interspecies transmission.

### Genome signature analysis and evolutionary trend

The pH1N1 influenza viruses circulating in Thailand still have typical hot spots [13]. Most were susceptible to oseltamivir (NA 275H) and zanamivir (NA 136S) but resistant to adamantane antiviral drugs. All isolates were able to synthesize a truncated PB1-F2 protein and seemed to have a normal rate of viral replication and transmission to mammalian host cells (PB2 627E). There was no significant difference of the mutation pattern of pH1N1 isolated from mild and severe cases. However, many amino acid signatures were found in different clades of virus. Clade 7, 8 and 11 pH1N1 isolates showed the mutation S220T of the HA gene. The mutation V731I, G154D of the PB1 gene and I330V mutation in the PA gene were predominantly found in pH1N1 isolates from clade 11.2. The A214T mutation in the HA gene was mainly found in isolates from clade 11, both 11.1 and 11.2. Almost all isolates circulating in clade 11 harbored the mutations S160G and S202T of the HA gene. A previous study has identified the genome signature of clade 8.1 as E391K of the HA gene, but in this study, this mutation was also present in pH1N1 isolates from clades 11.1 and 11.2. In this study, many evolutionary mutation trends were identified and have to be taken into consideration. The most conserved gene in genome signature analysis was M2 (Table 4 and Fig. 3). Considering the point mutations at S202T and S220T, highlighted as positions at one of the antigenic sites Ca [17], [25], it is unclear whether these amino acid signatures will effect a change in antigenic domain because these positions are not exposed to the surface and there was no other mutation of the amino acid signature at the Ca site. Likewise, analysis of HA diversity showed that the Thailand strain of pH1N1 still resembled the vaccine strain (CA/07/2009). Hence, the current vaccine is still effective to control pH1N1 infection in Thailand.

### Conclusion

Based on whole genome characterization, phylogenetic and genome signature analysis, it can be concluded that the circulation of pH1N1 influenza virus in Thailand during 2009–2012 has facilitated the virus establishing itself as a new seasonal influenza virus displaying gradual mutation and low pathogenicity. However, considering the health risk for patients with underlying conditions and pH1N1's capability of re-assortment, studies employing phylogenetic analysis and epidemiological surveillance have to be conducted to continuously monitor the evolutionary trend which will assist in preparations for and control of any future outbreak.

## Materials and Methods

### Ethical Consideration

The study was conducted on positive pH1N1 specimens collected from routine service and stored as anonymous. Permission had been granted by the Director of King Chulalongkorn Memorial hospital. Patient identifiers including personal information (name, address) and hospitalization number were removed from these samples to protect patient confidentiality and neither did they appear in any part of document in this study. The research protocol was approved by the Institutional Review Board (IRB number 284/54), Faculty of Medicine, Chulalongkorn University. IRB waived the need for consent because the samples were anonymous.

### Sample Collection

From May 4, 2009 until April 29, 2012, 11,753 nasal and throat swab samples were collected from 3 provinces, Bangkok, representing the central part of Thailand, Suraj Thani, representing the south and Khon Kaen, the north-east of Thailand. Samples were collected in viral transport media (VTM) from patients diagnosed with influenza-like illness (onset of high-temperature, fever, sore throat, or cough, watery eyes, body ache/headache and difficulty to breathe). RNA would be extracted from samples followed by real-time RT-PCR specific to influenza A, B and H1 (pandemic strain), H3 and H5 subtyping. [26], [27] The 40 pH1N1- positive specimens were selected randomly and chronologically (on average, 1–2 samples per month) from samples with a Ct value by real-time RT-PCR below 25 for primers specific for the M gene of influenza A and HA of pH1N1 influenza virus. In addition, approximate 200 pH1N1 positive specimens obtained in the course of the 3 waves of outbreak were also selected for significant specific mutation analyses. All specimens were kept at −70°C until RNA extraction.

### Viral RNA Extraction

RNA was extracted by using a commercially available Viral Nucleic Acid Extraction Kit (RBC Bioscience Co, Taiwan) according to the manufacturer's protocol. All experiments were performed in a Bio-safety Level 2 plus environment.

### Detection and Subtyping of Influenza Virus by Real-Time RT PCR

One-step multiplex real-time RT PCR assays were performed to detect and subtype influenza virus based on Taqman probes as previously described [26]–[28]. Each sample was subjected to 7 reactions testing for the GAPDH gene (internal control), M (matrix) gene of influenza A and B to determine type of influenza virus, HA (Hemagglutinin gene) for seasonal H1 and H3, H5 avian influenza virus and H1 of human pandemic influenza virus.

### Conventional PCR and Sequencing

Upon real-time RT-PCR, 40 samples positive for human pandemic influenza virus collected from three waves of outbreak in Thailand were chosen for whole genome characterization. Another 200 pH1N1 positive samples were selected to examine 6 genes for 7 point mutations defining the molecular characteristics of pH1N1 as previously described [13]. To begin with, viral cDNA was synthesized by using the M-MLV reverse transcription system (Promega, Madison, WI) and universal primers as described by Hoffman et al. [28] at 37°C for 3 hours. In brief, 1 µl of cDNA was added to the reaction mixture containing 15 µl of PerfectTaq Plus Mastermix (5Prime, Hamburg, Germany) with 0.5 µl of each forward and reverse primers, MgCl_2_ and nuclease free water to a final volume of 25 µl. PCR reactions were performed in a thermal cycler (Eppendorf, Germany) under the following conditions: Initial denaturation at 94°C for 3 minutes, followed by 40 amplification cycles comprising denaturation for 30 seconds, primer annealing at 50°C for 30 seconds (for PB1, PA, NP genes) and 55°C for 30 seconds (for PB2, HA, NA, M and NS genes) followed by extension at 72°C for 90 seconds, to be concluded by a final extension step at 72°C for 10 minutes. Upon electrophoresis in a 2% agarose gel, the amplified products were visualized on a UV trans-illuminator after staining with ethidium bromide solution. PCR products were purified using the HiYield Gel DNA Fragment Extraction kit (RBC Bioscience Co, Taiwan). DNA sequencing was performed by FirstBASE Laboratories Sdn Bhd (Selangor Darul Ehsan, Malaysia). The cDNA sequencing was performed by FirstBase Co.Ltd. (Kuala Lumpur, Malaysia). Primers used for whole genome characterization and hot spot analysis are shown in Table S1.

### Sequence analysis and Phylogenetic Tree Construction

DNAStar version 6 and Clustalw 1.83 were used for multiple sequence alignment. Bioedit7.0 was used to analyze the sequence alignment. The trimmed whole genome sequences were manually concatenated in sequence from segment 1–8 (PB2-PB1-PA-HA-NP-NA-M-NS) prior to phylogenetic tree construction. Reference strains for phylogenetic tree construction were retrieved from the NCBI influenza virus database based on a previous study [15]–[17]. MEGA 5.0 was used for the phylogenetic tree construction by applying the neighbor-joining method with Kimura's two-parameter distance model and 1000 bootstrap replicates [29]. BioEdit program version 7.0.9 was used for genomic signature analysis. The graphical presentation of relative residue abundance of genomic signatures within each gene was depicted by using WebLogo [30].

### Estimation of Nucleotide Substitution Rate and Measurement of Selection Pressure

The rates of evolutionary change in each gene (nucleotide substitution per site per year) were provided by using BEAST program version 1.4.6 [31]. The strict clock model was employed to provide the rate of nucleotide substitution. To evaluate the selection pressure on each gene segment of pH1N1 virus in Thailand, the mean values of non-synonymous substitution (dN) and synonymous substitution (dS) per site were calculated by using the single likelihood ancestor counting method (SLAC) based on Neighbor-Joining trees under the substitution model selected by the Datamonkey website (http://www.data-monkey.org).

## Supporting Information

Table S1 Primers used for whole genome characterization and hot spot analysis.
(DOCX)

Table S2 Accession numbers of 40 pH1N1 sequences in Thailand from 2009–2012 for whole genome characterization, phylogenetic tree construction and genomic signature analysis, separated by isolates.
(DOCX)

Table S3 Accession numbers of 584 pH1N1 sequences in Thailand for 7 major mutation analysis in 6 genes, separated by genes.
(DOCX)
