# Supplementary material for: Whole Genome Characterization, Phylogenetic and Genome Signature Analysis of Human Pandemic H1N1 Virus in Thailand, 2009–2012
Source: PLoS One. 2012 Dec 12;7(12):e51275. doi: 10.1371/journal.pone.0051275 (PMC3521005; doi:10.1371/journal.pone.0051275)
Supplement: Table S1 — Primers used for whole genome characterization and hot spot analysis. (DOCX) [file pone.0051275.s001.docx]

**Table s1:**

| **Primer Name** | **Nucleotide Sequences (5’🡪3’)** | **Tm (°C)** |
| --- | --- | --- |
| PB2_F5’ | 5’-AGCAAAAGCAGGTCAATTATATTC-3’ | 64 |
| PB2_R740 | 5’-CCTGGAGTGTACATCTGCTCCC-3’ | 64 |
| PB2_F496 | 5’-GTTGTTTTCCCAAATGAAGTGGG-3’ | 60 |
| PB2_R1253 | 5’-ATCGCCCCTAACTGCCTTGATCA-3’ | 62 |
| PB2_F832 | 5’-GCATCTCTCTTGGAAATGTG-3’ | 58 |
| PB2_R1823 | 5’-GCAAAGGGGAGAAGTTTTATTA-3’ | 60 |
| PB2_F1600 | 5’-TCAATGATGTGGGAGATCAATGG-3’ | 60 |
| PB2_F1683 | 5’-AGCAAAAGCAGGCAAACCAT-3’ | 60 |
| PB2_R3’ | 5’-AGTAGAAACAAGGTCGTTTTTAAAC-3’ | 66 |
| PB1_F5 | 5’-AGCAAAAGCAGGCAAACCATTTGAATGGATGTC-3’ | 80 |
| PB1_R520 | 5’-AATCTATTAGCCTTCCTGACTCAT-3’ | 66 |
| PB1_F711 | 5’- TGA ACA CRA TGA CCA ARG A -3’ | 50 |
| PB1_R843 | 5’-GTTCAAGCTTTTCRCAWATG-3’ | 60 |
| PB1_R1566 | 5’-AGCTCCATGCTRAAATTRGC-3’ | 60 |
| PB1_F1251 | 5’- TGAGTCCTGGAATGATGATG-3’ | 58 |
| PB1_R1863 | 5’- CCATTTCAAGCAGACTTCAG-3’ | 58 |
| PB1_F1705 | 5’- TGCCACAGAGGTGACACAC-3’ | 60 |
| PB1_R3’ | 5’-AGTAGAAACAAGGCATTTTTTCA-3’ | 60 |
| PA_F5’ | 5’-AGCAAAAGCAGGTACTGATCCG-3’ | 68 |
| PA_R613 | 5’-CTCTTTCGGACTGACGAAAG-3’ | 60 |
| PA_F361 | 5’-TATGAYTACAARGAGAA-3’ | 44 |
| PA_R989 | 5’-GGTTCTTTCCATCCAAAGAATGTT-3’ | 66 |
| PA_F894 | 5’-AAATTRAGCATTGAR GAYCCA -3’ | 54 |
| PA_R1662 | 5’-TCWAGTCTYGGGTCAGTGAG-3’ | 60 |
| PA_F1366 | 5’-ACTGAATACATAATAAGGG-3’ | 52 |
| PA_R3’ | 5’-AGTAGAAACAAGGTACTTTTTTGG-3’ | 64 |
| SWF_HA_F1 | 5’-AGCAAAAGCAGGGGAAAATAAAAGCA-3’ | 60 |
| SWF_HA_R694 | 5’-TCTTGATGACCCCACAAAAACATA-3’ | 58 |
| SWF_HA_F510 | 5’-AGCTTCTACARAAATTTAATATGGCT-3’ | 56 |
| SWF_HA_R1126 | 5’-CATCCATCTACCATCCCTGTCCA-3’ | 62 |
| SWF_HA_F1398 | 5’-GATTCAAATGTGAAGAACTTATATGA-3’ | 66 |
| SWF_HA_R1780 | 5’-AGTAGAAACAAAGGGTGTTTTTTCTCATGT-3’ | 62 |
| SWF_N1_F1 | 5’-AGCAAAAGCAGGAGTTCAAAATGAATC-3’ | 60 |
| SWF_N1_R575 | 5’-ATGACAAGCACTTGCTGACCAAG-3’ | 60 |
| SWF_N1_F437 | 5’-GCTAAATGACAAACATTCCAATGG-3’ | 58 |
| SWF_N1_R1115 | 5’-AATGCTTTTAGTTCTCCCTATCCA-3’ | 58 |
| SWF_N1_F957 | 5’-CAGATAGGATACATAAGCAGTGG-3’ | 60 |
| N1_R3’ | 5’-AGTAGAAACAAGGAGTTTTTTGAAC-3’ | 66 |
| NP_F5’ | 5’-AGCAAAAGCAGGGTAGATAATC-3’ | 62 |
| NP_R972 | 5’-GTGRGCTGGGTTTTCATTTGGTC-3’ | 68 |
| NP_F778 | 5’-GCACGGTCAGCACTYATCCTAAG-3’ | 58 |
| NP_R1205 | 5’-GCCCAGTATCTGCTTCTCA-3’ | 58 |
| NP_R3’ | 5’-AGTAGAAACAAGGGTATTTTTCT-3’ | 60 |
| SWF_M_F | 5’-AGCAAAAGCAGGTAGATATTTAAAGATGAGTCT-3’ | 68 |
| SWF_M_R | 5’-AGGTAGTTTTTTACTCYAGCTCTATGYTGACAA-3’ | 68 |
| M_R_3’ | 5’-AGTAGAAACAAGGTAGTTTTTTAC-3’ | 62 |
| NS_F5’ | 5’-AGCAAAAGCAGGGTGACAAAAAC-3’ | 66 |
| NS_R3’ | 5’-AGTAGAAACAAGGGTGTTTTTTAT-3’ | 62 |
| Uni_12_F | 5’- AGC AAA AGC AGG -3’ | 42 |
