# Supplementary material for: Whole Genome Characterization, Phylogenetic and Genome Signature Analysis of Human Pandemic H1N1 Virus in Thailand, 2009–2012
Source: PLoS One. 2012 Dec 12;7(12):e51275. doi: 10.1371/journal.pone.0051275 (PMC3521005; doi:10.1371/journal.pone.0051275)
Supplement: Table S2 — Accession numbers of 40 pH1N1 sequences in Thailand from 2009–2012 for whole genome characterization, phylogenetic tree construction and genomic signature analysis, separated by isolates. (DOCX) [file pone.0051275.s002.docx]

**Table s2:**

| **Name** | **Accession No.** |
| --- | --- |
| A/Thailand/nonthaburi102/2009 | CY040039 CY040040 CY040041 CY039986  CY039987 CY039988 CY039989 CY039990 |
| A/Thailand/104/2009 | GQ169381 GQ169382 GQ169383 GQ169384 GQ169385 GQ205443 GQ229379 GQ259597 |
| A/Thailand/CU-B5/2009 | GQ866948 [GQ866949](http://www.ncbi.nlm.nih.gov/entrez/viewer.fcgi?val=GQ866949) GQ866950 GQ866951 GQ866952 GQ866953 GQ866954 GQ866955 |
| A/Thailand/CU-H9/2009 | GQ866956 GQ866957 GQ866958 GQ866959 GQ866960 GQ866961 GQ866962 GQ866963 |
| A/Thailand/CU-H106/2009 | [GQ866928](http://www.ncbi.nlm.nih.gov/entrez/viewer.fcgi?val=GQ866928) GQ866932 [GQ866924](http://www.ncbi.nlm.nih.gov/entrez/viewer.fcgi?val=GQ866924) GQ866920 GQ866940 [GQ866944](http://www.ncbi.nlm.nih.gov/entrez/viewer.fcgi?val=GQ866944) GQ866916 GQ866936 |
| A/Thailand/CU-H276/2009 | GQ866945 GQ866941 GQ866925 GQ866917 GQ866921 GQ866929 GQ866933 GQ866937 |
| A/Thailand/CU-H340/2009 | GQ866918 GQ866922 [GQ866926](http://www.ncbi.nlm.nih.gov/entrez/viewer.fcgi?val=GQ866926) GQ866930 GQ866934 GQ866938 GQ866942 GQ866946 |
| A/Thailand/CU-C161/2009 | CY074971 CY074972 CY074973 CY074974 CY074975 CY074976 CY074977 CY074978 |
| A/Thailand/CU-H567/2009 | CY074987 CY074988 CY074989 CY074990 CY074991 CY074992 CY074993 CY074994 |
| A/Thailand/CU-H572/2009 | CY074995 CY074996 CY074997 CY074998 CY074999 CY075000 CY075001 CY075002 |
| A/Thailand/CU-H847/2009 | CY075003 CY075004 CY075005 CY075006 CY075007 CY075008 CY075009 CY075010 |
| A/Thailand/CU-H910/2009 | CY075011 CY075012 CY075013 CY075014 CY075015 CY075016 CY075017 CY075018 |
| A/Thailand/CU-C602/2010 | CY080304 CY080305 CY080306 CY080307 CY080308 CY080309 CY080310 CY080311 |
| A/Thailand/CU-B938/2009 | GQ866919 GQ866920 GQ866927 GQ866931 GQ866935 GQ866939 GQ866943 GQ866947 |
| A/Thailand/CU-H1222/2010 | CY074979 CY074980 CY074981 CY074982 CY074983 CY074984 CY074985 CY074986 |
| A/Thailand/CU-H1255/2010 | CY080312 CY080313 CY080314 CY080315 CY080316 CY080317 CY080318 CY080319 |
| A/Thailand/CU-H1818/2010 | CY080328 CY080329 CY080330 CY080331 CY080332 CY080333 CY080334 CY080335 |
| A/Thailand/CU-H1821/2010 | CY080336 CY080337 CY080338 CY080339 CY080340 CY080341 CY080342 CY080343 |
| A/Thailand/CU-H1786/2010 | CY080320 CY080321 CY080322 CY080323 CY080324 CY080325 CY080326 CY080327 |
| A/Thailand/CU-B2357/2010 | CY080296 CY080297 CY080298 CY080299 CY080300 CY080301 CY080302 CY080303 |
| A/Thailand/CU-B2417/2010 | CY088827 CY088828 CY088829 CY088830 CY088831 CY088832 CY088833 CY088834 |
| A/Thailand/CU-B2543/2010 | CY088835 CY088836 CY088837 CY088838 CY088839 CY088840 CY088841 CY088842 |
| A/Thailand/CU-H2176/2010 | CY088798 CY088799 CY088800 CY088801 CY088802 CY088803 CY088804 CY088805 |
| A/Thailand/CU-H2358/2010 | CY088813 CY088814 CY088815 CY088816 CY088817 CY088818 CY088819 CY082966 |
| A/Thailand/CU-H2283/2010 | CY088806 CY088807 CY088808 CY088809 CY088810 CY088811 CY088812 CY082965 |
| A/Thailand/CU-H2389/2010 | CY088820 CY088821 CY088822 CY088823 CY088824 CY088825 CY088826 CY082967 |
| A/Thailand/CU-H2911/2011 | CY089460 CY089461 CY089462 CY089463 CY089464 CY089465 CY089466 CY089467 |
| A/Thailand/CU-C1157/2010 | CY081154 CY081155 CY089442 CY081156 CY089443 CY081157 CY081158 CY081159 |
| A/Thailand/CU-H2548/2010 | CY089444 CY089445 CY089446 CY089447 CY089448 CY089449 CY089450 CY08951 |
| A/Thailand/CU-B4339/2010 | CY089434 CY089435 CY089436 CY089437 CY089438 CY089439 CY089440 CY089441 |
| A/Thailand/CU-B4148/2010 | CY089426 CY089427 CY089428 CY089429 CY089430 CY089431 CY089432 CY089433 |
| A/Thailand/CU-H2698/2010 | CY089452 CY089453 CY089454 CY089455 CY089456 CY089457 CY089458 CY089459 |
| A/Thailand/CU-B4656/2011 | **XXxxxxxx-** **XXxxxxxx** |
| A/Thailand/CU-B4662/2011 | **XXxxxxxx- XXxxxxxx** |
| A/Thailand/CU-B4717/2011 | **XXxxxxxx- XXxxxxxx** |
| A/Thailand/CU-B4773/2011 | **XXxxxxxx- XXxxxxxx** |
| A/Thailand/CU-B5356/2011 | **XXxxxxxx- XXxxxxxx** |
| A/Thailand/CU-B5515/2011 | **XXxxxxxx- XXxxxxxx** |
| A/Thailand/CU-B6181/2012 | **XXxxxxxx- XXxxxxxx** |
| A/Thailand/CU-B-6213/2012 | **XXxxxxxx- XXxxxxxx** |
