# Supplementary material for: Whole Genome Characterization, Phylogenetic and Genome Signature Analysis of Human Pandemic H1N1 Virus in Thailand, 2009–2012
Source: PLoS One. 2012 Dec 12;7(12):e51275. doi: 10.1371/journal.pone.0051275 (PMC3521005; doi:10.1371/journal.pone.0051275)
Supplement: Table S3 — Accession numbers of 584 pH1N1 sequences in Thailand for 7 major mutation analysis in 6 genes, separated by genes. (DOCX) [file pone.0051275.s003.docx]

**Table s3:**

| **Gene** | **Accession No.** |
| --- | --- |
| **PB2** | CY090129 CY090135 CY090141 CY090147 CY090153 CY090160 CY090166 CY090172 CY090178 CY090184 CY090190 CY090196 CY090203 CY090210 CY090217 CY090225 CY081106 CY081112 CY081118 CY081124 CY081130 CY081136 CY081142 CY081148 CY081160 CY075843 CY075670 CY075706 CY075682 CY075688  CY075694 CY075700 CY075706 CY075712 CY075718 CY075724 CY075730 CY075736 CY075742 CY075748 CY075754 CY075759 CY075765 CY075771 CY075777 CY075783 CY075789 CY075795 CY075801 CY075807 CY075813 CY075819 CY075825 CY075831 CY075837 CY081166 CY081172 CY081178 CY081184 CY081190  CY081196 CY081202 CY081208 CY081214 CY081220 CY087848 CY087854 CY087860 CY087866 CY087872 CY087878 CY087884 CY087890 CY087896 CY087902 CY087909 CY087915 CY087921 CY087927 CY087933 CY087939 CY087945 CY087951 CY087957 CY087963 CY090233 CY090241 CY090248 CY090255 CY090262  CY090270 CY090278 |
| **PB1** | CY090130 CY090136 CY090142 CY090148 CY090154  CY090161 CY090167 CY090173 CY090179 CY090185  CY090191 CY090197 CY090204 CY090211 CY090218  CY090226 CY081107 CY081113 CY081119 CY081125  CY081131 CY081137 CY081143 CY081149 CY081161  CY075743 CY075784 CY075671 CY075677 CY075683  CY075689 CY075695 CY075701 CY075707 CY075713  CY075719 CY075725 CY075731 CY075737 CY075749  CY075755 CY075760 CY075766 CY075772 CY075778  CY075790 CY075796 CY075802 CY075808 CY075814  CY075820 CY075826 CY075832 CY075838 CY075844  CY081167 CY081173 CY081179 CY081185 CY081191  CY081197 CY081203 CY081209 CY081215 CY081221  CY087849 CY087855 CY087861 CY087867 CY087873  CY087879 CY087885 CY087891 CY087897 CY087903  CY087910 CY087916 CY087922 CY087928 CY087934  CY087940 CY087946 CY087952 CY087958 CY087964  CY090234 CY090242 CY090249 CY090256 CY090263  CY090271 CY090279 |
| **HA** | CY090131 CY090137 CY090143 CY090149 CY090155  CY090162 CY090168 CY090174 CY090180 CY090186  CY090192 CY090198 CY090205 CY090212 CY090219  CY090227 CY081108 CY081114 CY081120 CY081126  CY081132 CY081138 CY081144 CY081150 CY081162  CY075672 CY075678 CY075684 CY075696 CY075702  CY075714 CY075720 CY075726 CY075738 CY075744  CY075761 CY075767 CY075773 CY075779 CY075785  CY075791 CY075797 CY075803 CY075809 CY075815  CY075821 CY075827 CY075839 CY075845 CY075690  CY075708 CY075732 CY075750 CY075756 CY075833  CY081168 CY081174 CY081180 CY081186 CY081192  CY081198 CY081204 CY081210 CY081216 CY081222  CY087850 CY087856 CY087862 CY087868 CY087874  CY087880 CY087886 CY087892 CY087898 CY087904  CY087911 CY087917 CY087923 CY087929 CY087935  CY087941 CY087947 CY087953 CY087959 CY087965  CY090235 CY090243 CY090250 CY090257 CY090264  CY090272 CY090280 |
| **NA** | CY090132 CY090138 CY090144 CY090150 CY090156  CY090163 CY090169 CY090175 CY090181 CY090187  CY090193 CY090199 CY090206 CY090213 CY090220  CY090228 CY081109 CY081115 CY081121 CY081127  CY081133 CY081139 CY081145 CY081151 CY081163  CY075673 CY075721 CY075745 CY075822 CY075679  CY075685 CY075691 CY075697 CY075703 CY075709  CY075715 CY075727 CY075733 CY075739 CY075751  CY075757 CY075762 CY075768 CY075774 CY075780  CY075786 CY075792 CY075798 CY075804 CY075810  CY075816 CY075828 CY075834 CY075840 CY075846  CY081169 CY081175 CY081181 CY081187 CY081193  CY081199 CY081205 CY081211 CY081217 CY081223  CY082964 CY087851 CY087857 CY087863 CY087869  CY087875 CY087881 CY087887 CY087893 CY087899  CY087905 CY087912 CY087918 CY087924 CY087930  CY087936 CY087942 CY087948 CY087954 CY087960  CY087966 CY090236 CY090244 CY090251 CY090258  CY090265 CY090273 CY090281 |
| **M** | CY090133 CY090139 CY090145 CY090151 CY090157  CY090164 CY090170 CY090176 CY090182 CY090188  CY090194 CY090200 CY090207 CY090214 CY090221  CY090229 CY081110 CY081116 CY081122 CY081128  CY081134 CY081140 CY081146 CY081152 CY081164  CY075674 CY075680 CY075686 CY075692 CY075698  CY075704 CY075710 CY075716 CY075722 CY075728  CY075734 CY075740 CY075746 CY075752 CY075763  CY075769 CY075775 CY075781 CY075787 CY075793  CY075799 CY075805 CY075811 CY075817 CY075823  CY075829 CY075835 CY075841 CY075847 CY075876  CY081170 CY081176 CY081182 CY081188 CY081194  CY081200 CY081206 CY081212 CY081218 CY081224  CY087852 CY087858 CY087864 CY087870 CY087876  CY087882 CY087888 CY087894 CY087900 CY087906  CY087908 CY087913 CY087919 CY087925 CY087931  CY087937 CY087943 CY087949 CY087955 CY087961  CY087967 CY090237 CY090245 CY090252 CY090259  CY090266 CY090274 CY090282 |
| **NS** | CY090134 CY090140 CY090146 CY090152 CY090158  CY090159 CY090165 CY090171 CY090177 CY090183  CY090189 CY090195 CY090201 CY090202 CY090208  CY090209 CY090215 CY090216 CY090222 CY090223  CY090224 CY090230 CY090231 CY090232 CY081111  CY081117 CY081123 CY081147 CY081153 CY081165  CY081135 CY081141 CY081129 CY075681 CY075687  CY075693 CY075705 CY075711 CY075717 CY075753  CY075776 CY075782 CY075788 CY075800 CY075836  CY075842 CY075848 CY075675 CY075699 CY075723  CY075729 CY075735 CY075741 CY075747 CY075758  CY075764 CY075770 CY075794 CY075806 CY075812  CY075818 CY075824 CY075830 CY075869 CY075870  CY075871 CY075872 CY075873 CY075874 CY075875  CY075877 CY075878 CY075879 CY075880 CY081171  CY081177 CY081183 CY081189 CY081195 CY081201 CY081207 CY081213 CY081219 CY081225 CY087853 CY087859 CY087865 CY087871 CY087877 CY087883 CY087889 CY087895 CY087901 CY087907 CY087914 CY087920 CY087926 CY087932 CY087938 CY087944 CY087950 CY087956 CY087962 CY087968 CY090238 CY090239 CY090240 CY090246 CY090247 CY090253 CY090254 CY090260 CY090261 CY090267 CY090268 CY090269 CY090275 CY090276 CY090277 CY090283 CY090284 CY090285 |
